# Rational Medicine; Assisting Nature

**Published:** 1897-01

**Authors:** 


### EDITORIAL DEPARTMENT.

F. E. DANIEL, M. D., Editor.
S. E. HUDSON, M. D., Managing Editor.
A. J. SMITH. M. D., Galveston, Associate Editor.

EDITORIAL STAFF:

PROF. J. E. THOMPSON, M. D., Texas Medical College, Galveston; Surgery.
PROF. WM. KEILLER, M. D., Texas Medical College, Galveston; Obstetrics and Gynecology.
PROF. DAVID CERNA, M. D., Texas Medical College, Galveston; Therapeutics.
PROF. A. J. SMITH, M. D., Texas Medical College, Galveston; Medicine.
DR. R. H. L. BIBB, City of Mexico; Foreign Correspondent.

Published Monthly at Austin, Texas, by Drs. Daniel and Hudson. Subscription price $2.00 a year in advance.

Eastern Agents: The Monihan-Fairchild Special Agency, 24 Park Place, New York City.

Official organ of the West Texas Medical Association, the Houston District Medical Association, the Austin District Medical Society, Brazos Valley Medical Association, the Galveston County Medical Society, and several others

### RATIONAL MEDICINE; "ASSISTING NATURE."

For the first time in the history of medicine, the practice may be said to be based upon rational principles.

In the light of recent discoveries,—dating from that of the function of the white blood corpuscle,—which led to a conception first, and then a demonstration of the *modus operandi* of what has, for long ages, been understood as the *vis medicatrix naturæ;* that certain cells do police duty, and others perform the function of scavengers, while the avenues of approach to the vital seat are guarded at every portal by infinitesimal sentinels, endowed apparently with a life intelligence, we see and realize that all that has gone before was, for the greater part empirical: and that, guided by the false lights of the "humoral" and other absurd pathologies which preceded the bacterial age, the practice of medicine had nothing solid to stand upon, and was based, almost solely, on a few facts which long experience and observation had taught, but of the reason for which facts no cause was known, nor even a rational conjecture offered. For instance: it was known that a wound would heal better under a scab than if left open; and therefore, it was easily learned to exclude the air, but without knowing why; that quinine would cure chill and fever, without a conception of the *modus operandi*, nor, really, of the cause of the paroxysms, the *plasmodium malariæ*, and the fact that quinine is its "antitoxin," are recent discoveries. Now, cause and effect being well understood, the administration of quinine for malaria may be said to be rational. The great fact discovered and demonstrated by Jenner was empirical; not a conception was had,—hardly a conjecture hazarded to explain why vaccination with the lymph of kine-pox would protect from small-pox. In light of subsequent knowledge, following the discovery of living cells in the blood, and that each set or species have a specific office, it has been learned to assist nature intelligently, by seconding as it were,—her efforts, for instance—in throwing off effete matter. Hence "stimulating the emunctories" became not only the most popular but the most efficacious form of medication,— and hence, too,—one may say—the great demand for and popularity of purgative pills. The era is now dawning when the assistance to nature assumes a more intelligent and rational aspect.

The study of bacteriology, by slow degrees, unraveled the mystery of fermentation, and led to the discovery of toxines, or the products of bacilli, in the blood, and the manner in which it is antagonized in the living laboratory readily suggested artificial antitoxine. Hence the plan of fighting specific diseases upon the same principles that "nature" fights against them; and with this knowledge the rational practice now is, to reinforce nature, to strengthen the army of occupation and defense; to supply to the system an antitoxine manufactured in the laboratory. Already a number of deadly diseases have succumbed to this new weapon of science. Pasteur, Koch and Haffkine have been the benefactors of this generation, and have given the world a rational therapeusis. The latter's success with cholera-inoculation naturally suggested the idea of supplying to the blood artificial antitoxines that are found in the blood in other diseases, as antagonistic to the germs of that disease; and the latest triumph claimed for the new therapeutics is

PROTECTIVE INOCULATION AGAINST TYPHOID FEVER.

According to the N. Y. *Medical Journal*, and other leading journals that have access to the German periodical medical literature, Prof. R. Pfeiffer and Dr. W. Kolle, of the Berlin Institute for Infectious Diseases, have been experimenting with the blood of typhoid fever patients, aiming to determine the practicability of protecting against typhoid fever by injecting a product of the dead bacilli of that disease. For their experiments they choose persons with a clear history of never having had typhoid fever. The first injection caused reaction within two or three hours; rigors, giddiness, a feeling of malaise, and pain at the site of the puncture. The evening temperature rose to about 101.3° F., and the first night's restwas somewhat disturbed. The temperature on the following morning was still somewhat elevated, but soon fell to normal, and all symptoms ceased.

The New York *Medical Journal* says:

"The author concludes that one injection of a very small amount of the agent brings about a specific change in the blood which is recognizable after six days, and reaches at least as high a grade as they have ever been able to observe in convalescents from the disease. They think it more than probable that the presence of specific substances destructive of bacteria in the blood of persons who have had typhoid fever accounts for the immunity that is known to be enjoyed by such persons. If this assumption is correct, they say, prophylactic inoculations with the dead culture may be expected to confer an immunity of the same degree and duration as occurs after the disease has been incurred in the natural way, and they hold that their position is justified by the results of Haffkine's analogous protective inoculations against cholera, which have stood the severest test in many thousand cases." * * The hope is expressed that these protective inoculations against typhoid fever may become of practical value under certain conditions; for example, in the face of severe epidemic, for they may be managed easily and speedily by any medical practitioner without special preparation, provided, he has at hand the material in proper condition."

Attention is called in the same article, to the many trials of typhoid fever cultures and their derivatives by Brieger, Wassermann and Frankel, but it is remarked that they were not for prophylaxis, but in the treatment of the disease.

What with small-pox, cholera, diphtheria and typhoid fever disarmed of their power to destroy human life; with the knowledge that the disease can not only be defeated, arrested in mid career perhaps, but absolutely prevented, and with the means of preventing it in one's possession—man will have but little to fear in the way of acute disease.

Now, when science can record a similar victory over consumption—and the work of Maragliano shows rapid progress toward the accomplishment of that end*, we see no reason why man should not, in nearly every instance, live out his scriptural measure of years, and reach the sere and yellow leaf in the enjoyment of his three score and ten,—barring accidents,—and thus, with the life average greatly lengthened, the cost of life insurance ought to be much lessened.

Science, in the treatment of the human machine, has attained almost the perfection of the watchmaker who, looking into the delicate works of a piece of fine mechanism, detects at once the cause of stoppage or irregularity, and applies the remedy. When it has become possible as it is, almost, now—to take a drop of blood, analyze it and tell what it contains that is detrimental to the human system, and eliminate it, or detect what is missing and supply it; to know that after recovery from a specific disease there is, in the blood, a substance antagonistic to bacteria—and, *ergo*, that to put such substances into the blood of a well person will give him immunity; or to actually look into, and through a living person; to see the pulsations of the heart; to see the blood circulating in the capillaries by means of the wonderful X-rays, one asks what more is lacking to enable man to make life perpetual? It begins to look as if the key to the problem of life has been found. Vaccination was a hint, the significance of which the world has been more than a hundred years in interpreting.

---

State Health Officer's Report: The report of the State Health Officer for the years 1895-96 has just been issued from the office of the State printers. It is a pamphlet of some thirty pages, and embraces a report of quarantine and internal sanitation, together with other matters pertaining thereto.

---

*Dr. Zaeslein, of Geneva, (*New York Medical Journal*, August 15, 1896, The Serum Treatment of Pulmonary Tuberculosis) remarks that "the serum treatment consists essentially in introducing into the human body substances, which, either of themselves oppose the germ of the disease or lead to the formation of such materials (antitoxins) in the organism. Animals have been treated with the tuberculous matter in order to engender large amounts of antitoxins in their blood. For this purpose Maragliano used cultures of the tubercle bacillus, but without living bacilli. The serum of animals systematically treated with this material annuls the action of tuberculine. The writer states further that Maragliano's serum has passed the experimental stage, and may safely be received into practical therapeutics."

